# What Causes Environmental Inequalities and Related Health Effects? An Analysis of Evolving Concepts

**DOI:** 10.3390/ijerph110605807

**Published:** 2014-05-30

**Authors:** Hanneke Kruize, Mariël Droomers, Irene van Kamp, Annemarie Ruijsbroek

**Affiliations:** 1 Centre for Sustainability, Environment and Health (DMG), National Institute for Public Health and the Environment (RIVM), Antonie van Leeuwenhoeklaan 9, 3721 Bilthoven, The Netherlands; E-Mail: Irene.van.Kamp@rivm.nl; 2 Department of Public Health, Academic Medical Centre, University of Amsterdam, Meibergdreef 9, 1012 WX Amsterdam, the Netherlands; E-Mail: m.droomers@amc.uva.nl; 3 Centre for Nutrition, Prevention and Health Services, National Institute for Public Health and the Environment (RIVM), Antonie van Leeuwenhoeklaan 9, 3721 Bilthoven, The Netherlands; E-Mail: Annemarie.Ruijsbroek@rivm.nl

**Keywords:** environmental justice, environmental inequalities, health inequalities, conceptual framework, integrated approach, multilevel

## Abstract

Early environmental justice studies were exposure-oriented, lacked an integrated approach, and did not address the health impact of environmental inequalities. A coherent conceptual framework, needed to understand and tackle environmental inequalities and the related health effects, was lacking. We analyzed the more recent environmental justice literature to find out how conceptual insights have evolved. The conceptual framework of the WHO Commission on Social Determinants of Health (CSDH) was analyzed for additional explanations for environmental inequalities and the related health effects. This paper points out that recent environmental justice studies have broadened their scope by incorporating a broader set of physical and social environmental indicators, and by focusing on different geographic levels and on health impacts of environmental inequalities. The CSDH framework provided additional elements such as the role of structural determinants, the role of health-related behavior in relation to the physical and social environment, access to health care, as well as the life course perspective. Incorporating elements of the CSDH framework into existing environmental justice concepts, and performing more empirical research on the interactions between the different determinants at different geographical levels would further improve our understanding of environmental inequalities and their health effects and offer new opportunities for policy action.

## 1. Introduction

At the Fifth Ministerial Conference on Environment and Health in Parma in 2010, the Member States of the WHO European Region committed to act on socio-economic and gender differences in health that are a consequence of environmental inequalities. It was stated that ‘the existence of significant unjust and avoidable inequalities in environmental risks within a country is not acceptable, and… calls for relevant policies and interventions’ [1]. The question is what is the most effective way to tackle these environmental inequalities? To answer that question, we need insight into the underlying mechanism.

Environmental inequalities are the focus of the so-called “environmental justice” domain. Environmental justice consists of two dimensions. First of all, it refers to the spatial distribution of environmental risks and amenities and the resulting disparities among socio-economic and racial groups (“distributional” or “geographical justice”). It includes all places, *i.e.*, where people live, but also where they work, learn, play, and recreate. Second, it refers to the distribution process itself, including access to and participation in decision-making processes and procedures that create environmental risks (“procedural justice”). The lack of both distributive justice and procedural justice often characterizes socio demographically disadvantaged groups [1].

The environmental justice debate started in the 1960s in the USA and was empowered by activists. The debate initially focused on local issues, but became a national issue in the USA in the 1990s, after the environmental justice movement had been established. Furthermore, several influential studies had appeared, indicating that minorities and those with lower incomes were unequally exposed to environmental pollutants. For example, research from the US-EPA pointed out that minorities and those with lower incomes were exposed more often to several air pollutants, hazardous waste facilities, contaminated fish, and agricultural pesticides at the workplace. In addition, black children had significantly higher blood lead levels compared to white children [2]. Most of the many studies published in the years thereafter confirmed that minorities and low-income groups were indeed disproportionately exposed to environmental hazards [3,4,5].

Although the vast majority of environmental justice studies has been performed in the USA, other countries have paid attention to the issue of environmental justice as well, including Australia [6,7], Canada [8,9], and South Africa [10]. Over the past decade, it has also received increasing attention in Europe, in particular in the United Kingdom [11,12,13,14,15], and more recently in Italy, France, the Netherlands, and Germany [16,17,18,19,20,21,22]. Like the US studies, these European studies generally show higher residential environmental risks for less affluent populations [18,19,21].

Most of the aforementioned studies solely describe environmental inequalities—differences in exposure to environmental risks and in access to amenities—rather than explaining them. Insight in the mechanisms behind these inequalities, however, is needed to be able to tackle them effectively. The few studies that described the mechanisms stem from different disciplinary fields, such as geography, sociology, and economy. Kruize [17] summarized these mechanisms described in the studies that were published before 2004. She took Liu’s multidisciplinary overview of existing theories as a point of departure [23]. In short, the studies state that the spatial distribution of environmental risks and amenities among socioeconomic and racial groups mainly resulted from the combination of the location of polluting activities and residencies [1]. Industries, for example, locate where land prices are low and labor forces reside. Therefore people with a lower socio-economic status often live nearby these industries. Furthermore, the distribution of power may also contribute to environmental inequalities. Geographically remote, economically marginal, and/or politically powerless communities and their residents tend to lack effective political power and the ability or will to influence or resist decisions that affect their living environment. Last, some authors refer to institutionalized racism in housing and planning practices as an explanation for environmental inequalities [17].

The early environmental justice studies that try to explain environmental inequalities encountered a number of limitations. First, a coherent conceptual framework, in which insights from the aforementioned disciplinary angles are integrated, was lacking. By applying a specific disciplinary angle, they can only partly explain environmental inequalities. Second, most of these studies focused on the community or neighborhood level, while other levels (societal, individual) may drive inequalities as well. Third, they often focused on a single pollutant rather than on the accumulation of environmental pollutants, while unfavorable social, spatial, and environmental conditions may accumulate in certain areas. Fourth, most studies did not pay attention to the health impacts of differential environmental exposure for different socio-economic groups—the so-called environmental health inequalities. This may be explained by the fact that the environmental justice domain developed from the exposure-oriented environmental justice movement. Furthermore, datasets that enable an assessment of variations of environmental exposure and the related health effects are lacking. Nevertheless, attention for the health effects of the unequal distribution of environmental risks and amenities among the population is important, since it is not just the difference in exposure that matters, but the fact that these differences contribute to health inequalities [17]. Moreover, people with a low socio-economic status may be more vulnerable to environmental exposures since they often are in poorer health. Consequently, health effects due to environmental exposures may be more severe and occur at lower levels of exposure in people with a low socio-economic status compared to the general population [1].

This paper aims to find out how the concepts regarding environmental inequalities have evolved during the last decade and if the aforementioned limitations have been overcome. To do so, we analyzed the more recently developed conceptual models in the environmental justice domain and described their added value. Second, we analyzed the conceptual framework of the WHO Commission on Social Determinants of Health (CSDH) for additional explanations for environmental health inequalities. Many researchers in the public health domain have tried to explain health inequalities for years and their insights could help to explain environmental inequalities [24]. With our analysis of the literature we aim to enhance our understanding of environmental inequalities and the related health effects, and in that way contribute to more effective ways to tackle these inequalities.

## 2. Methods

To analyze the more recently developed concepts and theories in the environmental justice domain, Medline was searched for publications in English between January 2004 and July 2013. Concepts and theories to explain environmental inequalities published before 2004 have been summarized by Kruize and others [17]. The MeSH terms on (synonyms of) environmental justice were combined with those regarding health and health inequalities and (synonyms of) theories (Table 1). MeSH terms are Medical Subject Headings (MeSH). These are comprehensive controlled vocabulary for the purpose of indexing journal articles and books in the life sciences. This resulted—after deleting doublings—in a list of 336 papers.

**Table 1:** Literature research profile for recent environmental justice concepts and theories.

1 *Environment/(17258) 2 *Environmental Health/(8187) 3 *Environmental Exposure/(23148) 4 *Environmental Pollution/(6860) 5 1 or 2 or 3 or 4 (53890) 6 Health Status/or Healthcare disparities/(64031) 7 *social class/or *social conditions/or *socioeconomic factors/or (social inequalit* or social inequit* or socioeconomic* or disparit*).ti. (35029) 8 exp Socioeconomic Factors/(329076) 9 6 or 7 or 8 (382457) 10 (theory or theories or mechanism* or concept* or model* or pathway* or explanat* or framework*).mp. (4383149) 11 (theory or theories or mechanism* or concept* or model* or pathway* or explanat* or framework* or analys* or measure* or eviden*).ti,kw. (1682481) 12 *Models, Theoretical/(38355) 13 *Research/or Systems theory/(119340) 14 *Epidemiologic Methods/(5717) 15 11 or 12 or 13 or 14 (1817642) 16 (environmental adj3 (health or disparit* or justice or equit* or inequit* or inequalit* or racism or deprivat* or profil*)).ti. (3394) 17 16 and 11 (253) 18 (environmental adj3 (health or disparit* or justice or equit* or inequit* or inequalit* or racism or deprivat* or profil*)).mp. (19819) 19 (18 or 5) and 9 and 15 (477) 20 17 or 19 (687) 21 limit 20 to year = 2004–2 July 2013 (373) ^1^

**Figure 1:**
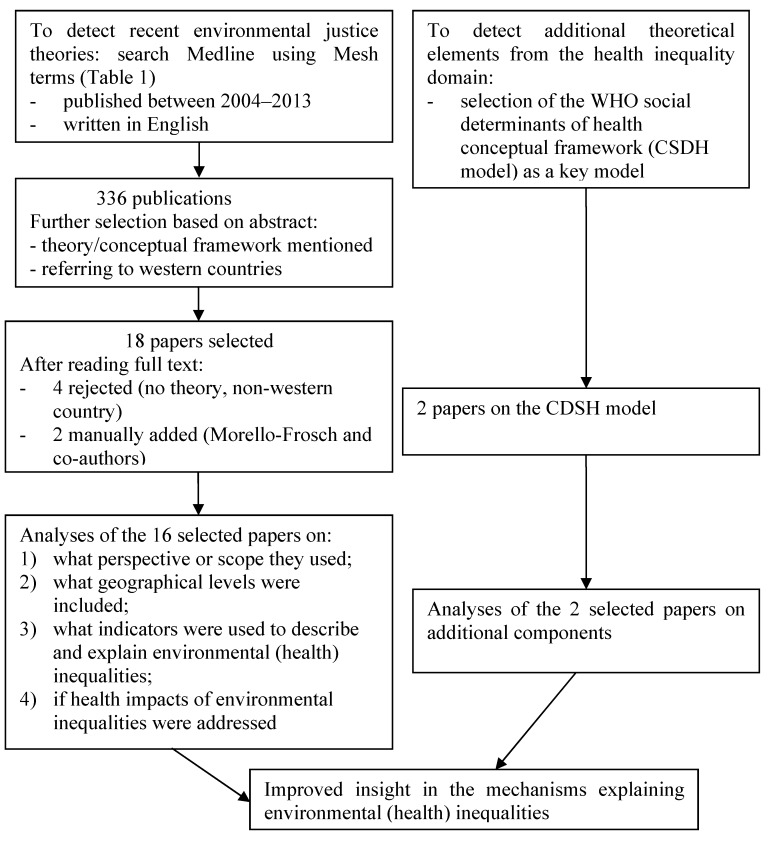
Flowchart on methodology of the pragmatic review on concepts and theories to explain environmental inequalities.

^1^ The difference between the total number of references presented in this table and in Figure 1/method section can be explained by some doublings in references.

The abstracts of these papers were screened using the following inclusion criteria:

(1) they should mention a conceptual framework or theory regarding environmental inequalities;
(2) they should refer to environmental inequalities in western countries.

Eighteen papers met these criteria. Four papers were rejected after reading the full text. These papers did not include a conceptual framework or theory and/or referred exclusively to the situation in developing countries, although their abstracts indicated otherwise. Two papers of Morello-Frosch and co-authors were mentioned in several of the selected papers, but did not show up in the search, so we added them to the selection [25,26]. The resulting sixteen studies were analyzed with regard to elements related to lacking concepts and theories in the earlier environmental justice papers, in particular:

(1) What perspective or scope they used;
(2) What geographic levels—community, neighborhood, other—were included;
(3) What indicators were used to describe and explain environmental (health) inequalities;
(4) If health impacts of environmental inequalities were addressed.

To detect additional conceptual elements from the health inequality domain, we analyzed the WHO CSDH framework, being a key framework that integrates the theoretical insights on health inequalities. We studied two key publications describing the CSDH framework [24,27] and compared the components from this framework with the recent environmental justice concepts retrieved from the Medline search. For that we did not use the four elements (scope, geographical level, attention for set of indicators, health inequalities) on which we analyzed the more recent environmental justice studies. Figure 1 summarizes the methodology used.

## 3. Results and Discussion

### 3.1. Developments in Concepts to Explain Environmental Inequalities from the Environmental Justice Domain

Table 2 provides an overview of the more recently developed concepts and theories in the environmental justice domain. Since most of these papers describe concepts rather than theories, we refer to concepts and conceptual frameworks in our analyses. We describe the results around the four key issues outlined in the “Methods” section, pertaining to the scope of the paper, geographical levels included, indicators used to describe and explain environmental (health) inequalities, and health impacts addressed.

#### 3.1.1. A broader, More Integrated Scope

While many of the earlier environmental justice studies described socio-economic disparities for one environmental pollutant, the more recent conceptual papers often have a more integrated, system-oriented approach, paying attention to the context of environmental inequalities. Several use a multiple or cumulative risk assessment approach, in which they focus on multiple (sources of) risks [2,5,6,8]. Some went one step further. Morello-Frosch and Shenassa used a holistic approach focusing on the interplay of environmental hazards with place-based and individual level psychosocial stressors [26]. Gee and Payne-Sturges used a transdisciplinary approach. These authors developed a transdisciplinary scientific foundation to explore the conceptual issues, data needs, and policy applications to measure and track environmental inequalities and related health effects, together with people working in universities, community organizations, and state and federal agencies to develop [28]. Still, most of the papers use the exposure-disease paradigm as a starting point for their conceptual work.

#### 3.1.2. Multiple Geographical Levels

In the more recent conceptual frameworks and theories on environmental justice, multiple levels are distinguished. Soobader *et al.* distinguish the macro or institutional level, the local neighborhood or community level, and the micro or individual level [29], while others make a distinction between community and individual level [26,30].

**Table 2:** Characteristics of the selected environmental justice papers.

	Authors	Aim	Scope/hypothesis	Geographical level(s) included	Indicators used to describe and explain environmental health inequalities	Addresses health impacts?
1.	Bolte, G., *et al.* (2009) [18]	Evaluating evidence on environmental inequalities among children in Europe and discussing policy implication	Socio-economic divide, differential environmental conditions, differential vulnerability, health outcomes, health services, access and differential quality	International, national, local	traffic-related air pollution, noise, lead, environmental tobacco smoke, indoor air pollution, housing/built environment (including impact on physical activity), water pollution, waste.	Yes, but main focus is differential environmental exposure
2.	Burger, J.; Gochfeld, M. (2011) [31]	Presenting a conceptual model for evaluating nonstandard, unique, or excessive exposures	Source, pathway, route of exposure, receptor	Individual/population	airborne pollutants, dermal exposures, pollutants in food & water (ingestion), medicinal exposures (injections)	No; paper focuses on exposures
3.	Crowder, K.; Downey, L. (2010) [32]	Examining the extent and sources of environmental inequality at the individual level	Patterns and determinants of individual proximity to industrial pollution, in particular residential mobility; roles of economic conditions and racial barriers in residential mobility	Individual and neighborhood	proximity to industrial pollution, education, income, age, marital status, number of children, home ownership, household crowding, length of residence	No; focus on proximity to industrial pollution
4.	Cutts, B.B., *et al.*(2009) [33]	Evaluating the relationship between the distribution of populations vulnerable to obesity and proximity to parks and walkable street networks	The built environment shapes both behavior and health outcomes: more walkable neighborhoods and access to parks correlate with higher levels of physical activity and lower body mass index (BMI)	Neighborhood	(1) local park access, (2) walkable neighborhoods, social modifiers: (a) traffic speed and (b) traffic fatality rates, (c) crime rates, and (d) park size as proxies for differences in residents’ perception of quality of the built environment.	No
5.	DeFur, P.L., *et al.* (2007) [34]	Examining the issue of vulnerability in cumulative risk assessment and presenting a conceptual framework	Uses a cumulative risk assessment approach. Health outcomes are predicted by the relationships among measures of the (chemical, physical, biological and social stressors), receptor characteristics (measures of potential vulnerability) and receptor resources (abilities to respond or recover).	Community, population, individual	Environmental, social, biological, psychosocial	No
6.	Evans, G.W.; Kim, P. (2010) [35]	Examining whether multiple risk exposure could account in part for the SES-health gradient	Multiple risk exposure is considered as a mediating mechanism for social gradients in health, with attention for lifetime exposure, both at home and at the workplace.	Community, individual	Physical and psychosocial indicators:Housing and neighborhood quality, pollutants and toxins, crowding and congestion, and noise Adverse interpersonal relationships with family members, friends, supervisors, and community members. Counts of stressful life events	Yes, (perceived) health, morbidity and mortality
7.	Gee, G.C.; Payne-Sturges, D.C. (2004) [30]	Providing a multidisciplinary framework to understand how social processes may interrelate with environmental toxicants, and to understand why some groups experience greater illness compared with other groups	Stress-exposure-disease framework. Starting point is the exposure-disease paradigm Residential segregation is considered as a driver for structural factors at community level physical and psychosocial risks and resources, resulting in community stress. That affects individual stress, depending on individual susceptibility and coping strategies.	Community, individual	Community level: Structural factors (e.g., local economy) Physical and psychosocial risks Neighborhood resources Individual level: Coping strategy Susceptibility	Yes, health effects of stress
8.	Linder, S.H.; Sexton, K. (2011) [36]	Examining why decisions about theoretical frameworks matter for cumulative risk assessment, and identifying 3 families of conceptual models to understand and estimate combined health risks from environmental, social, and psychological factors.	Focuses on cumulative risk assessment. Including social determinant models, health disparity models, and multiple stressors models	Macro/society Community Individual	Many different indicators; see Figure 1–6 in Linder and Sexton (2011) for more details	Yes, focuses on models from both the environmental and public health domain
9.	Morello-Frosch,R.; Lopez, R (2006) [25]	Examining theoretical and methodological questions related to racial residential segregation and environmental health Disparities	Uses the lens of racial residential segregation to reveal whether observed pollution—health outcome relationships are modified by segregation and whether segregation disproportionately impacts certain populations. Builds further on existing concepts (Gee and Payne-Sturges, 2004, a.o.)	Macro/societal Community Individual	Macro level: Structural mechanisms of discrimination. Residential segregation Community level: Indicators of the built and social environment Individual level: Social support, income, poverty, working conditions, educational status, diet-nutritional status, psychosocial stress, health behaviors	Yes
10.	Morello-Frosch, R.; Shenassa, E.D. (2006) [26]	Presenting evidence that individual-level and place-based psychosocial stressors may compromise host resistance such that environmental pollutants would have adverse health effects at relatively lower doses, thus partially explaining MCH disparities, particularly poor birth outcome	Uses a holistic approach, focusing on interplay of environmental hazards with place-based and individual level psychosocial stressors and its implications for research on maternal and child health. Starting point is the exposure-disease paradigm	Community Individual	Stressors and buffers of the built and social environment Individual level stressors and buffers	Yes, birth outcomes
11.	Payne-Sturges, D.; Gee, G.C. (2006) [37]	Discussing one potential tool, a set of candidate measures that may be used to track disparities in outcomes, as well as measures that may be used analytically to assess potential causal pathways	States that health disparities are partly created by differential access to resources and exposures to hazards. Categories of indicators: social processes, environmental contaminants/exposures, body burdens of environmental contaminants, and health outcomes.	National/macro (Available measures/indicators) Community Individual (framework)	Social processes: Residential segregation Community stressors Neighborhood resources Structural factors Physical environment hazards Outdoor/indoor air pollution Drinking water/ambient water quality Pesticides Land contaminants and waste sites (Table 1, p. 158/159)	Yes, body burden, mortality, chroming diseases, infectious diseases, children’s health
12.	Payne-Sturges, D., *et al.* (2006) [28]	Developing a transdisciplinary scientific foundation for exploring the conceptual issues, data needs, and policy applications associated with social and environmental factors used to measure and track racial, ethnic, and class disparities in environmental health.	Uses a transdisciplinary approach, using the stress-exposure disease (SED) framework of Gee and Payne-Sturges (2004) as a starting point	National/macro Community Individual	Upstream social and environmental factors identified for selected health outcomes (Table 3, p. 150)	Yes, broad set of health outcomes (Table 2 of paper, p. 149)
13.	Soobader, M., *et al.* (2006) [29]	Proposing a multilevel conceptual framework for environmental health inequalities	Uses a multilevel approach	Macro/Society Local/community Micro/individual	No extensive list; focus is on importance of multilevel approach	Yes
14.	Stafford, M., *et al.* (2007) [38]	Theorizing a model of the potential causal pathways to obesity and employing path analysis	States that features of the local social and physical environment may affect obesity through encouraging physical activity and through promoting healthy eating	Environmental/ community Individual	Contextual level: Measures of local infrastructure and services (e.g., high street facilities) Measures of neighborhood socio-relational characteristics (e.g., neighborhood disorder) Individual level: Age, gender, SES	Yes, takes health as a starting point
15.	Taylor, W.C., *et al.* (2007) [39]	Reviewing “first Wave” (early work) of the environmental justice (EJ) movement, presenting second wave (“more recent work”) of the EJ movement, discussing implications of adopting principles from the EJ movement to focus on research in parks and recreation services (PRS), and recommending future research directions.	States that unequal access to physical activity facilities and resources (e.g., parks, recreational facilities) among socio-economic and racial groups may contribute to differences in physical activity and obesity	Community	Parks and recreational facilities	Yes, physical activity and obesity
16.	Van Kamp, I., *et al.* (2004) [40]	Reviewing conceptual and methodological issues regarding health differences at the neighborhood level. Evaluating theoretical public health and environmental health approaches in their ability to overcome such problems	Uses an integrated approach on health differences at the neighborhood level	Neighborhood Individual	Environmental (physical and social): natural environment, natural resources, built environment, public services, accessibility social environment/community, culture, *etc.* Individual: genes, personality, behavior/habits, health, lifestyle, economic position, motives, preferences, *etc.* Both community personal level: social capital and social networks	Yes

##### Macro or Institutional Level

The macro level refers to both the larger geospatial region (e.g., states, counties) that includes several communities. It also refers to the broader social context (e.g., political climate, environmental laws/enforcements, national economy) [29,30], which may contribute to residential segregation [25]. Residential segregation—the spatial separation of the residences of racial and income groups from one another—is a central element in the more recent environmental justice conceptual frameworks [26,30,34]. Residential segregation can be considered as an outcome of several factors already mentioned in the earlier environmental justice literature, such as economic changes, institutionalized discriminatory practices in the housing market, or preferences of residents to cluster together [25]. DeFur *et al.* state that residential segregation shapes all institutions, affecting the quality of schools, homes, transportation, commercial facilities, and safety and security [34]. According to Morello-Frosch and Lopez it shapes the distribution of resources and wealth at the individual and community level, and may result in differential exposure to environmental risks between socio-economic groups [25]. Gee and Payne-Sturges pointed out that segregation is not solely negative. They stated that segregation may create “supportive social relationships within minority communities that may help promote health and well-being and ameliorate the effects of community risks” (p. 1649) [30].

##### Community or Neighborhood Level

A broad set of environmental and psychosocial risks, produced by the both the physical and social environment, accumulate in neighborhoods with a lower socio-economic status [25,30]. Next to risk factors, resources or amenities are also present at the neighborhood level, such as political power and supportive social relationships. These may reduce the negative effects of the risks. In Section 3.1.3 we elaborate upon this process.

##### Individual Level

Several of the more recent studies also pay attention to individual differences, in particular with regard to the response to environmental exposures [26,30,34]. Disadvantaged groups are more vulnerable to adverse health effects of environmental exposures than the general population due to differences in health status and biological sensitivity [1]. Vulnerability in this paper refers to “how individuals or groups of individuals or organisms respond to and recover from stressors inadequately or not as well as the average” ([34], p. 817). In addition, personal coping resources as well as possibilities to control negative impacts from environmental exposures determine whether, and to what extent environmental exposures have health consequences. For example, the perception that one can regulate the degree of negative environmental circumstances can have profound positive effects on both psychological and physiologic health outcomes [34]. Social support, the physical health status may influence an individual’s coping resources [30]. Moreover, having less access to information on the effects of environmental exposures may also affect their coping strategies regarding environmental exposures [1]. In case an individual is not able to deal with negative environmental exposures for a long time period, this may result in chronic stress. This can have long-term health consequences and lead to immune dysfunction [34]. Furthermore, chronic stress may render individuals more susceptible to illness when exposed to environmental risks [26,30].

#### 3.1.3. Indicators to Describe and Explain Environmental Inequalities and Related Health Effects

##### A Broad Set of Physical and Psychosocial Environmental Risks

Most of the selected papers recognize that people with a lower socio-economic status are exposed to a much broader range of environmental risks than mentioned in the earlier environmental justice studies [30]. These risks are produced by the physical and social environment and include not only physical indicators (e.g., noise, temperature, radiation), but also psychosocial indicators, such as crowding, social disorganization, racial discrimination, fear of e.g., crime, and economic deprivation [29,30,34]. Burger and Gochfeld broadened the set of indicators in a different direction by focusing on different pathways of exposure to environmental pollutants (inhalation, dermal, ingestion, and injection) to address nonstandard vulnerabilities, unique pathways, and behaviors that lead to excessive exposures and disproportionately high environmental health risks [31].

##### Neighborhood Resources

Next to risk factors, the social environment also generates resources or amenities, such as political power and supportive social relationships, also called neighborhood resources. Empowered communities may be better able to resist unwanted land use developments, such as the location of a polluting industry. Furthermore, socially cohesive groups may undertake collective action more often, enhancing a feeling of collective control. In these ways neighborhood resources may reduce the negative effects of the aforementioned environmental risks. If these resources cannot outweigh the effects of the environmental risks, community stress—a state of ecological vulnerability—will manifest. This may lead to individual stress and subsequent illness [30].

#### 3.1.4. Addressing Health Impacts of Environmental Inequalities

The more recent studies in the environmental justice domain pay explicit attention to the health impacts of environmental inequalities in different ways [30,37,39]. Gee and Payne-Sturges describe the health effects of stress that may result from environmental exposures [30]. Taylor *et al.* refer to the consequences of differential access to parks and other recreational facilities on physical activity, an important determinant of health [37].

### 3.2. The CSDH Conceptual Framework

We analyzed the conceptual framework of the WHO CSDH for additional explanations for environmental health inequalities. This rich framework is based on a review and summary of the (conceptual) knowledge on the social determinants of health [24]. The CSDH distinguishes two levels of determinants of health inequalities. The first level includes the structural drivers—institutions and processes of the socioeconomic and political context that create social hierarchies. Based on their socioeconomic position, individuals experience differences in exposure and vulnerability to conditions that may have a negative effect on their health—the second level of causation. The main categories of intermediary determinants of health are:

- Material circumstances, including housing and neighborhood quality, financial means to buy healthy food, warm clothing, *etc.*, and the physical work environment;
- Psychosocial circumstances, including psychosocial stressors, stressful living circumstances and relationships, and social support and (lack of) coping styles;
- Behavioral and biological factors. Behavioral factors include diet, physical activity, tobacco consumption and alcohol consumption. Biological factors also include genetic factors;
- The health system, including access to health care and health promotion.

For a more detailed description of the CSDH conceptual framework we refer to the publication of Solar and Irwin [24]. There are a number of similarities between the more recent environmental justice conceptual frameworks and the CSDH framework, which we describe in the next section (3.2.1). Additions to the existing environmental justice concepts and theories to explain environmental health inequalities derived from the CSDH framework are described in Section 3.2.2.

#### 3.2.1. Similarities between Recent Environmental Justice Concepts and the CDSH Framework

We see several similarities between the CSDH framework [24] and the more recent environmental justice concepts, in particular, Gee and Payne-Sturges [30] and DeFur *et al.* [34]. First, all use an integrated approach, placing inequalities in a broader context to understand how these inequalities are created. Second, both the more recent environmental justice concepts and the CDSH framework recognize the importance of a multilevel approach, with determinants at the macro (national), meso (community), and micro (individual) level that contribute to inequalities [24,29]. The CSDH introduced an additional level—the global level. The studies that appeared from our literature search did not focus on this level, although a specific stream of earlier environmental justice studies as described by e.g., Kruize, did focus on the international level, for example in terms of hazardous waste dumping, climate change, biodiversity, and natural resources [17]. Third, both the CSDH framework and several of the more recent environmental justice papers ([30,34], a.o.) describe the role of the social and physical environment as determinants of both stressors and resources—although using different wording. The “material circumstances” mentioned in the CSDH framework are strongly linked with the physical environment as referred to in the environmental justice studies, although “material circumstances” in the CDSH framework have a broader scope and also contain, for example, the financial means to buy healthy food and presence of stores that sell healthy food. Similarly, the psychosocial circumstances of the CSDH framework are comparable with the stressors and neighborhood resources mentioned by Gee and Payne-Sturges [30] and DeFur *et al.* [34].

Next to these similarities, there are also a number of topics mentioned in the CSDH framework that have not appeared in the environmental justice concepts, but may prove to be valuable additions, *i.e.*, the role of structural drivers at the macro level, health related behavior or lifestyle, the role of the health sector, and the life course perspective.

#### 3.2.2. More Extensive Consideration of Structural Drivers

The CDSH framework considers structural drivers more extensively than most of the environmental justice conceptual frameworks. Structural drivers as included in the CDSH framework comprise the social, economic, and political mechanisms that create socioeconomic stratification, including the labor market, the educational system, political institutions and other cultural and societal values (p. 5) [24], as well as policies that may redistribute welfare. In some environmental justice studies structural drivers are recognized as well (e.g., [25]). However, they have a different meaning, since they mainly refer to the community level and largely overlook the role of structural mechanisms at the societal level [29].

#### 3.2.3. Health Related Behavior or Lifestyle

The CSDH framework also brings forward behavioral or lifestyle factors as relevant intermediary determinants of health status. These include diet, physical activity, and tobacco and alcohol consumption. The social and physical environment influences this behavior [24]. For example, tobacco consumption and alcohol consumption may increase in a stressful social environment. A second example is that physical exercise may be stimulated by an attractive and accessible physical environment. Behavior is therefore a relevant pathway by which the environment affects health.

#### 3.2.4. Role of the Health Sector

The health sector may be of relevance for the environmental justice domain in different ways. First, the health sector aims to improve people’s health, also by treating health consequences of environmental exposures. Healthier people are assumed to be less susceptible to environmental exposures, and have more options to secure their social status. Offering people equal access to health care of equal quality creates equal chances for people to improve their health, and therefore being less susceptible to environmental exposures. 

Indirectly, the health sector can affect environmental health inequalities by empowering people—e.g., by providing information on the potential health effects of their living environment—or by offering them social support [24]. Furthermore, the health sector may press the “upstream” sectors such as transport, housing, the economic and the environmental sector to pay attention to the health consequences of their policies and actions as well as to inequalities that may result from it [1].

#### 3.2.5. Life Course Perspective

Most environmental justice studies try to explain environmental inequalities at a certain moment in time, although more recently some apply a life course perspective [26,35,40]. The CDSH, however stresses the importance of a life course perspective explicitly. This perspective “recognizes the importance of time and timing in understanding causal links between exposures and outcomes within an individual life course, across generations, and in population-level diseases trends” (p. 18) [24]. Factors operating early in life course may have implications for disease outcomes in adulthood [24]. Applied to environmental health inequalities, exposure to certain social and physical neighborhood environments during childhood may make people more vulnerable for diseases, affecting health later in their life. For example, exposure during a specific period in life course may have lasting or lifelong effects on the structure or function of organs, tissues, and body systems [24]. In addition, there may be an increasing cumulative damage to biological systems when exposures, e.g., to air pollution, accumulate over the life course. The life course perspective makes clear that it is important to consider environmental exposures lifelong.

### 3.3. Discussion

Based on our analyses of the selected literature, we conclude that many of the limitations of the earlier environmental justice studies have been overcome in the more recent conceptual papers. They have adopted a broader scope, by using a multilevel approach, by adopting a broader set of indicators, and by addressing the health consequences of environmental inequalities. Furthermore, we find that the conceptual framework of the WHO CSDH contains valuable additional elements for the environmental justice domain. First, the role of structural drivers—social, economic, and political mechanisms—in the production of environmental inequalities is recognized. Second, health related behavior or lifestyle may mediate the relation between the environment and health inequalities. Third, the health sector plays a role in reducing (environmental) health inequalities, since this sector may reduce differences in exposures, in vulnerability, and in the consequences of illness for people’s health. Last, we learned that it is important to consider environmental exposures across the life.

Before elaborating upon our findings, we need to address some limitations of our analyses of the literature. First, it is possible we have missed some recent conceptual papers in the environmental justice domain. The environmental justice domain is a multidisciplinary field. Therefore, a number of relevant papers may not have been captured by searching Medline. For example the papers of Morrello-Frosch and co-authors [25,26] did not show up from our literature search, but was referred to by others several times. Second, there may be other frameworks on health inequalities that may provide additional elements to explain environmental health inequalities that were not included in the CDSH framework. Linder and Sexton, for example, distinguish three “families” of conceptual models or theoretical frameworks on cumulative risks: (i) social determinant models (e.g., CDSH model); (ii) health disparity models, and (iii) multiple stressors models [29]. Each family “shares the same theoretical roots” (p. S75) [36]. The overview of Linder and Sexton provides a good overview of these frameworks, the overlap, and differences. We refer to their paper for more details [36], and acknowledge the fact that we might have missed additional elements that are dealt with exclusively in the health disparities or multiple stressors models. Third, we started this review from the view that the environmental justice domain and the public health domain are still two separate domains. However, it appeared that these domains have grown closer to each other and partly overlap. Several of the selected environmental justice papers focus on public health topics [33,38,39]. For example, Taylor *et al.* presented a framework on the effect of access to parks and recreational facilities on physical activity under the denominator of environmental justice [39]. What we have selected as an environmental justice publication could therefore in some occasions also have been considered a public health publication. However, for the understanding of what causes environmental inequalities and its related health effects this distinction is not relevant, as becomes clear in this paper. On the contrary, we state that a further integration of the conceptual frameworks from the environmental and health domain seems to be a fruitful way forward to enhance our understanding of environmental inequalities and the related health impacts [24,28,29,40].

In order to take a next step into the direction of a coherent conceptual framework to improve our understanding of environmental inequalities and the related health effects, we synthesize the conceptual elements we distilled from our analyses of the literature, using a multilevel approach. At the *macro or societal level*, social, economic, and political mechanisms create socioeconomic stratification [24] and residential segregation [25]. Residential segregation is considered an important determinant of environmental inequalities, since it shapes all institutions and in that way affects the quality of e.g., schools, homes, and transportation [25]. At the *community and neighborhood level,* both the physical and social environment produce a broad set of environmental and psychosocial risks that accumulate in neighborhoods with a lower socio-economic status. The environment also generates resources [29]. It is remarkable that the studies we reviewed mainly focused on resources of the social environment, while the physical environment also offers health enhancing resources. The social and physical environment also influences people’s health related behavior [24]. For example, creating accessible healthy public spaces (e.g., green space) may have a positive effect on health related behavior of all people [24], regardless of their socio-economic status [41,42]. At the *micro or individual level*, differences in vulnerability and in coping strategies contribute to differential health impacts of the environment. If an individual is not able to deal with negative environmental exposures for a long period, this may result in chronic stress. This can have long-term health consequences and lead to immune dysfunction [34]. Furthermore, chronic stress may render individuals more susceptible to illness when exposed to environmental risks [26,29]. Improving people’s health and empowering them may make them less susceptible to environmental exposures. The health sector as well as other “upstream sector” play an important role in this [1,24]. Last, we learned from the CDSH framework that it is not only important to study the described determinants at multiple levels, but also consider environmental exposures lifelong as proposed in the life course approach [24].

This multilevel interpretation is to a certain extent comparable to an approach used by WHO to define action in order to reduce environmental inequalities [43,44]. In his editorial Braubach states that action should focus on (1) societal structures and mechanisms that cause or contribute to environmental inequalities at the macro level; (2) the resulting disparities in environmental exposures existing at the community or neighborhood level; and (3) the potential vulnerability differences existing at the individual level [43]. These three entry points for action are also distinguished in the priority public health conditions analytical framework developed by the Priority Public Health Conditions Knowledge Network of WHO to investigate how health equity could be improved in the first place through public health programs. Two additional entry points are mentioned in the framework at the individual level, namely differential health outcomes and social and economic consequences for the individual level [44].

In the above we attempted to integrate the conceptual “pieces of the puzzle” to enhance our understanding of environmental inequalities and the related health impacts. To develop these ideas further, empirical studies are needed. As yet, there is only limited empirical insight in the interactions between these drivers at different levels [45], but also regarding individual drivers in relation to environmental inequalities. For example, the mechanism by which psychosocial stress increases individual and community vulnerability and affects health, is not fully clear yet, and needs further attention [45]. Moreover, empirical insights on environmental exposures in the course of the life are lacking, due to the complexities of measuring and characterizing neighborhood environments over the life course [40,45].

A main difficulty in empirical testing is the lack of data [35]. Environmental exposure data are often collected and reported without reference to race, ethnicity, social class or gender, which impedes the description of disparities and the analysis of their potential drivers [37]. Moreover, even in case these socio-demographic data are available in environmental studies, they are often not linked to health data. Furthermore, available data are often derived from cross-sectional studies, making it hard to assess causal associations. There is a clear need for longitudinal studies to track health conditions and risk factors [25,29,37,45]. Evaluation of the impact of experiments may provide additional insights, but may be complex or not feasible for a number of reasons, such as the need for a large number of neighborhoods [45]. Additionally, it may be difficult to unravel effects at different levels such as the neighborhood and individual level due to spatial interdependencies. Another complication is that it is not fully clear yet at what levels environmental risks and resources, socio-economic status, and health should be studied [37].

Last, our understanding of environmental inequalities would be greatly improved if multiple risk factors and health impacts would be measured over the life, for example to provide insight in susceptible developmental phases, but also in accumulated of risks as a person matures [35].

It is important to find solutions for these empirical problems and data needs, since it hampers the further development of a coherent conceptual framework, needed to further improve our understanding of environmental inequalities and the related health effects. This is crucial for the search for effective ways to tackle these inequalities.

## 4. Conclusions

The concepts described in the more recent environmental justice papers use a broader, more integrated, multilevel approach, and pay more explicit attention to health effects than the earlier studies. Hereby, they have largely overcome the limitations of the earlier concepts and theories. Further integration of environmental justice concepts with insights from the public health domain is a promising way forward, because it enhances our understanding of environmental inequalities and the related health effects. A general challenge of all work on environmental justice is that more empirical research is needed, in particular into the interactions between the different determinants and geographical levels, requiring longitudinal environmental, health, and socio-demographic data. This could further improve our understanding of environmental inequalities and the related health effects and offer new opportunities for policy action.
